# Data on cytochrome *c* oxidase assembly in mice and human fibroblasts or tissues induced by *SURF1* defect

**DOI:** 10.1016/j.dib.2016.03.065

**Published:** 2016-03-26

**Authors:** Nikola Kovářová, Petr Pecina, Hana Nůsková, Marek Vrbacký, Massimo Zeviani, Tomáš Mráček, Carlo Viscomi, Josef Houštěk

**Affiliations:** a Institute of Physiology of the Czech Academy of Sciences, Vídeňská 1083, Prague, Czech Republic; b Molecular Neurogenetics Unit, Instituto Neurologico “C. Besta”, via Temolo 4, 20126 Milan, Italy; c MRC-Mitochondrial Biology Unit, Wellcome Trust MRC Bldg, Addenbrookes Hospital Hills Rd, Cambridge CB2 0XY, UK

**Keywords:** COX, Cytochrome *c* oxidase, DOX, doxycycline, Cytochrome *c* oxidase, Respiratory chain, SURF1, Knockout, Doxycycline

## Abstract

This paper describes data related to a research article entitled “Tissue- and species-specific differences in cytochrome *c* oxidase assembly induced by *SURF1* defects” [1]. This paper includes data of the quantitative analysis of individual forms of respiratory chain complexes I, III and IV present in *SURF1* knockout (*SURF1*^*−/−*^) and control (*SURF1*^*+/+*^) mouse fibroblasts and tissues and in fibroblasts of human control and patients with *SURF1* gene mutation. Also it includes data demonstrating response of complex IV, cytochrome *c* oxidase (COX), to reversible inhibition of mitochondrial translation in *SURF1*^*−/−*^ mouse and *SURF1* patient fibroblast cell lines.

**Specifications Table**

**Table:** 

Subject area	Biochemistry
More specific subject area	Mitochondria, COX assembly, SURF1 protein
Type of data	Figures
How data was acquired	Western blots of SDS and BNE/SDS PAGE, antibody signals quantification, values expressed in percent of controls.
Data format	Analyzed, presented in text
Experimental factors	*SURF1* mouse knockout, human *SURF1* mutations, doxycycline inhibition of mitochondrial DNA translation
Experimental features	Digitonin solubilization of mitochondrial proteins, immunodetection of respiratory chain complexes
Data source location	Department of Bioenergetics, Institute of Physiology, Czech Academy of Sciences, Czech Republic, Prague
Data accessibility	Data are provided in this article

**Value of the data**

• Different proportions and native forms of respiratory chain complexes detected by 2D PAGE and WB in mammalian tissues or cells.
• Tissue- and species-specificity of COX biogenesis at normal and pathological conditions.
• Reversible mitochondrial translation arrest for analysis of newly synthesized COX in mouse/human fibroblasts.
• Approach to study different assembly defects of respiratory chain complexes containing mtDNA-encoded subunits.

## 1 Data

**Fig. 1:**
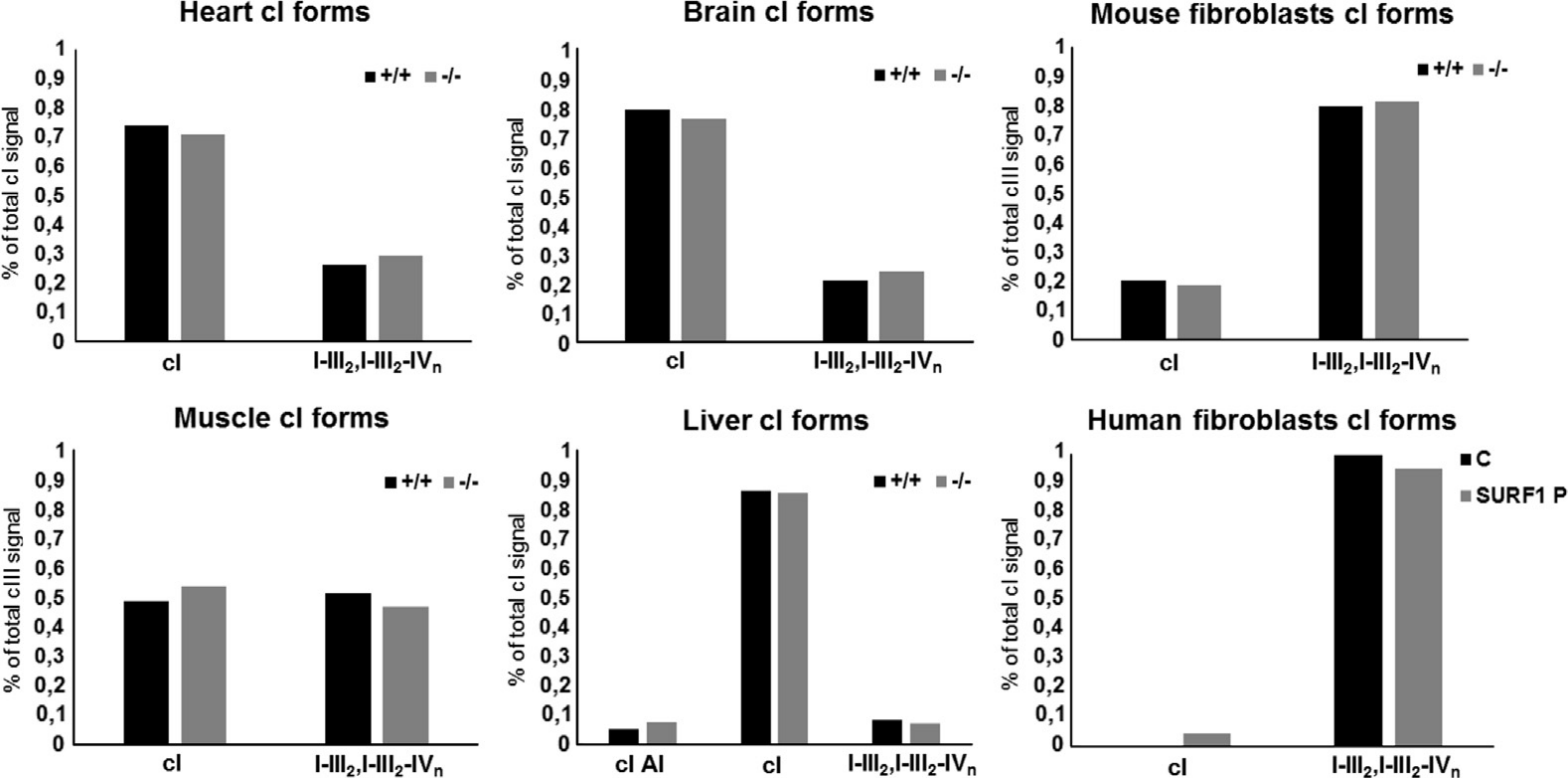
Complex I (cI) forms present in *SURF1*^*−/−*^ mouse tissues and fibroblasts and *SURF1* patient fibroblasts. For analysis, *SURF1*^*+/+*^ mouse (+/+), *SURF1*^*-/-*^ mouse (−/−), human control (C) and *SURF1* patient (SURF1 P) data from BNE/SDS PAGE western blots (see Fig. 1 in [1]) were used. Signals of NDUFB6 (NDUFS3 in muscle) subunit were quantified and expressed as percentage of overall NDUFB6 (NDUFS3) signal of each tissue/cell western blot. cI assembly intermediates (cI AI); supercomplexes I–III_2_ and I–III_2_–IV_n_ (I–III_2_, I–III_2_–IV_n_).

**Fig. 2:**
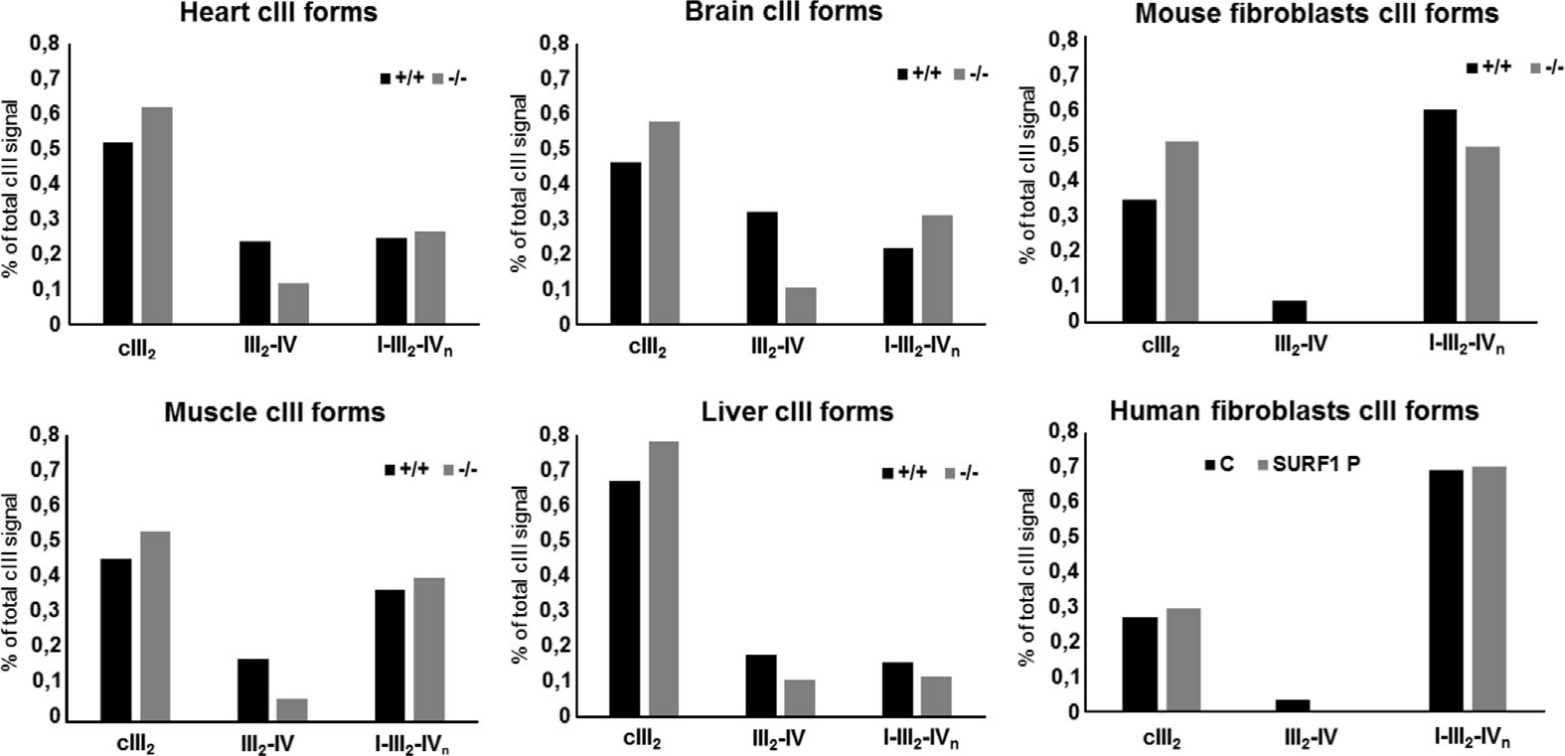
Complex III (cIII) forms present in *SURF1*^*–/–*^mouse tissues and fibroblasts and *SURF1* patient fibroblasts. For analysis, *SURF1*^*+/+*^ mouse (+/+), *SURF1*^*−/−*^ mouse (−/−), human control (C) and *SURF1* patient (SURF1 P) data from BNE/SDS PAGE western blots (see Fig. 1 in [1]) were used. Signals of CORE1 subunit were quantified and expressed as percentage of overall CORE1 signal of each tissue/cell western blot. cIII dimer (cIII_2_); supercomplexes III_2_–IV, I–III_2_ and I–III_2_–IV_n_ (III_2_–IV, I–III_2_, I–III_2_–IV_n_).

**Fig. 3:**
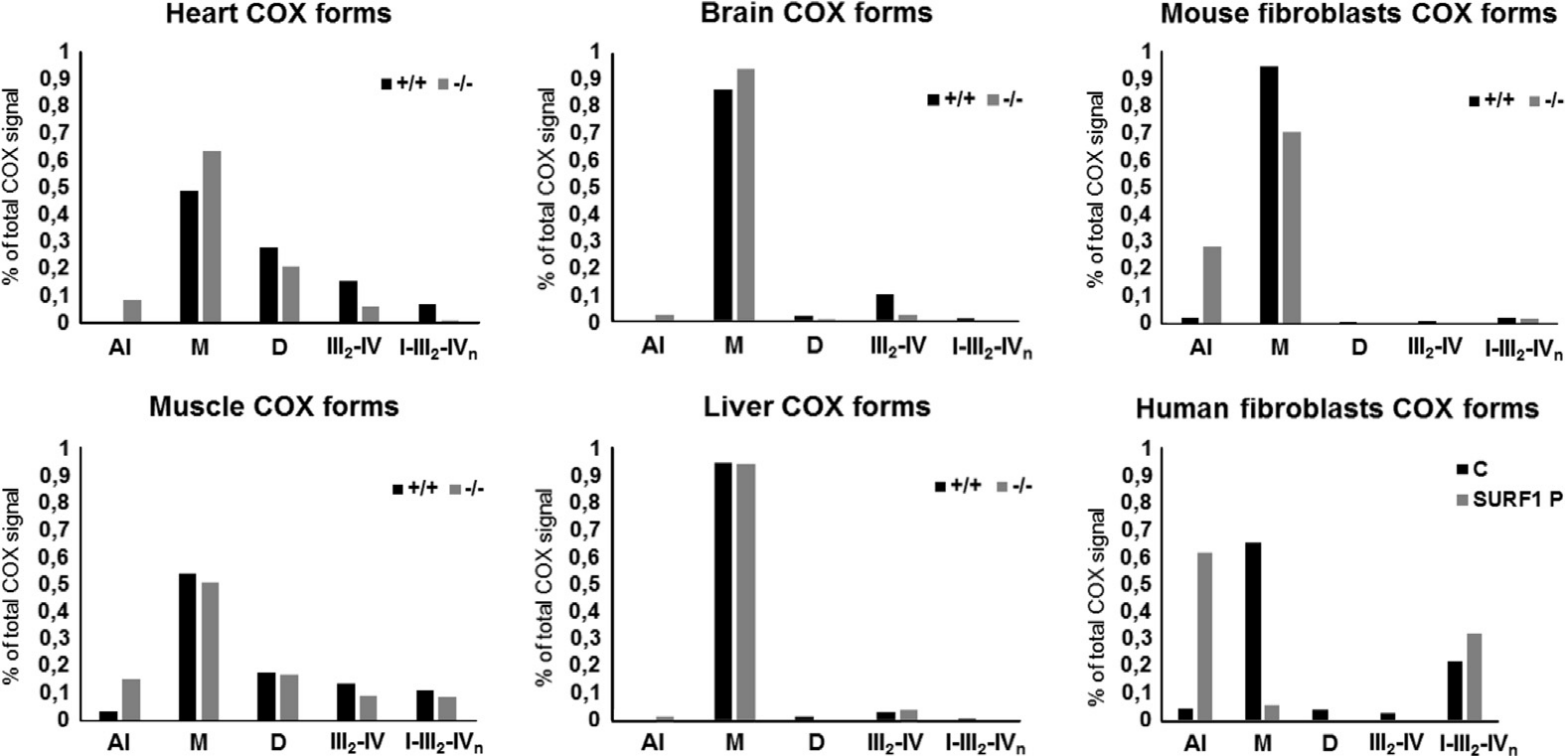
COX forms present in *SURF1*^*−/−*^mouse tissues and fibroblasts and *SURF1* patient fibroblasts. For analysis, *SURF1*^*+/+*^ mouse (+/+), *SURF1*^*−/−*^ mouse (−/−), human control (C) and *SURF1* patient (SURF1 P) data from BNE/SDS PAGE western blots (see Fig. 1 in [1]) were used. Signals of COX1 were quantified and expressed as percentage of overall COX1 signal in each tissue/cell western blot. COX assembly intermediates (AI), COX monomer (M), COX dimer (D), supercomplexes III_2_–IV and I–III_2_–IV_n_ (III_2_–IV, I–III_2_–IV_n_).

In the present work related to [1], we show differences in amounts of individual forms of respiratory chain complexes I, III and IV quantified from western blots of 2D BNE/SDS PAGE analysis, as determined in mitochondria of *SURF1*^*+/+*^ and *SURF1*^*−/−*^ mouse fibroblasts and tissues (heart, muscle, brain, liver) and also in mitochondria of human control and SURF1 deficient fibroblasts (Fig. 1, Fig. 2, Fig. 3).

**Fig. 4:**
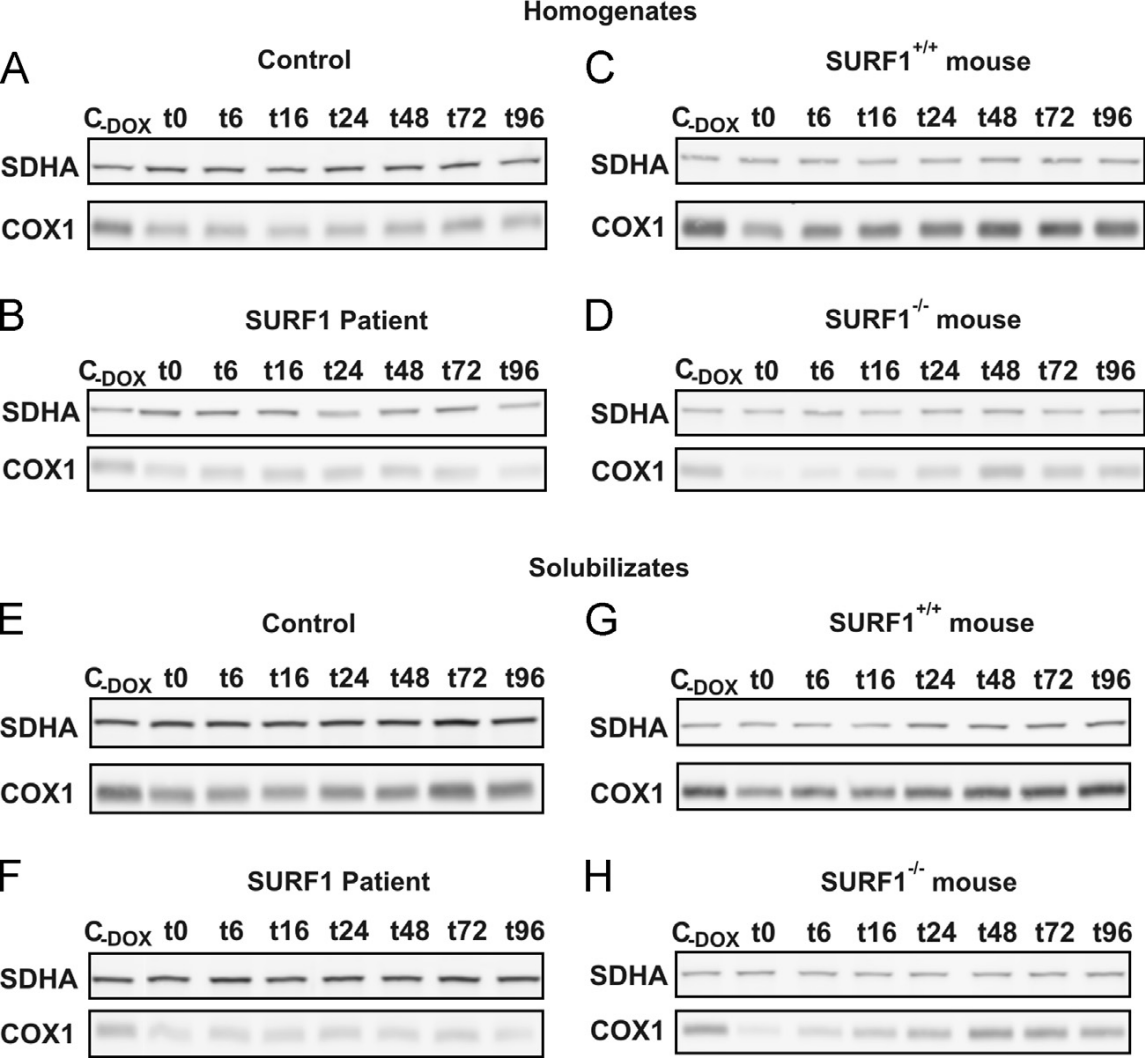
Decreased COX1 amount after DOX treatment. Homogenates from DOX treated human control and *SURF1* patient fibroblasts (A, B), *SURF1*^*+/+*^ and *SURF1*^*−/−*^ mouse fibroblasts (C, D) and digitonin solubilizates from DOX treated human control and *SURF1* patient fibroblasts (E, F), *SURF1*^*+/+*^ and *SURF1*^*−/−*^ mouse fibroblasts (G, H) were analyzed on SDS PAGE in combination with western blot to obtain overall COX1 signal at different time points (0–96 h) after DOX treatment. Signal of SDHA was used as reference. Control cells without DOX treatment (C_-DOX_).

Then we show data (Fig. 4) from analysis of fibroblast cell lines from *SURF1*^*−/−*^mouse, *SURF1* patient and controls, in which translation of mitochondrial DNA encoded proteins was reversibly inhibited with doxycycline (DOX). After DOX removal, the formation of newly synthetized COX in time (0–96 h) was assessed by SDS PAGE and western blot analysis.

## 2 Experimental design, materials and methods

### 2.1 Experimental material

For experiments different tissues were obtained from 3-month old *SURF1*^−/−^ knockout B6D2F1 mice [2], generated by the insertion of a *loxP* sequence in exon 7 of the mouse *SURF1* gene, leading to an aberrant, prematurely truncated and highly unstable protein, and from control wild type *SURF1*^+/+^ mice. Immortalized skin fibroblasts from control and *SURF1*^*−/−*^ mouse were cultured at 37 °C in 5% atmosphere of CO_2_ in a DMEM medium supplemented with 10% fetal bovine serum, 20 mM HEPES (pH 7.5) and geneticin (50 µg/ml). The same conditions were used for cultivation of human patients’ skin fibroblasts lacking the SURF1 protein due to 845 del CT mutations of *SURF1* gene [3] and from controls, except that geneticin was replaced with penicillin (10 µg/ml) and streptomycin (10 µg/ml). The project was approved by the ethics committees of Institute of Physiology, CAS. Informed consent was obtained from the parents of the patients according to the Declaration of Helsinki of the World Medical Association.

### 2.2 Isolation of mitochondria and cell membranes

Muscle (hind leg) was minced in a K medium (150 mM KCl, 2 mM EDTA, 50 mM Tris, pH 7.4) supplemented with protease inhibitor cocktail (1:500, PIC from Sigma) and homogenized by ultra turrax IKA (2x for 15 s, level 4) and glass-teflon homogenizer (600 rpm, 5 strokes). 5% (w/v) homogenate was centrifuged 10 min at 600*g* and postnuclear supernatant was centrifuged 10 min at 10,000*g*. Pelleted mitochondria were washed once (10,000*g*, 10 min) and resuspended in K medium.

Liver mitochondria were isolated from 10% homogenate prepared in STE medium (250 mM sucrose, 10 mM Tris, 2 mM EDTA, pH 7.2) supplemented with PIC (1:500) using glass-teflon homogenizer (600 rpm, 7 strokes). The homogenate was then centrifuged for 10 min at 800*g*. Postnuclear supernatant filtered through a gauze was centrifuged for 15 min at 5200*g*, pelleted mitochondria were washed twice (13,000*g*, 10 min) in STE with PIC and then resuspended in STE medium.

Heart mitochondria were isolated essentially as liver mitochondria, except that postnuclear supernatant was centrifuged for 10 min at 13,000*g*.

Fibroblast mitochondria were isolated according to Bentlage et al. [4] with slight modifications. Cells harvested using 0.05% trypsin and 0.02% EDTA were sedimented (600*g*, 5 min) and washed twice in phosphate-buffered saline (PBS − 140 mM NaCl, 5 mM KCl, 8 mM Na_2_HPO_4_, 1.5 mM KH_2_PO_4_, pH 7.2). Weighed cell pellet was suspended in 10 times (w/v) the amount of 10 mM Tris-buffer with PIC (1:500) and homogenized by teflon-glass homogenizer (8 strokes, 600 rpm). Immediately afterwards 1/5 volume of 1.5 M sucrose was added. Homogenate was centrifuged at 600*g*, 10 min and mitochondria containing supernatant was kept on ice. Pellet was suspended in original volume of SEKTP (250 mM sucrose, 40 mM KCl, 20 mM Tris, 2 mM EGTA, pH 7.6, PIC 1:500), rehomogenized (5 strokes, 800 rpm) and centrifuged at 600*g*, 10 min. The supernatants were pooled and centrifuged at 10,000*g*, 10 min. Sedimented mitochondria were washed with SEKTP (10,000*g*, 10 min) and suspended in SEKTP.

Frozen cell pellets were resuspended in sucrose buffer (83 mM sucrose, 6.6 mM imidazole/HCl, PIC 1:500, pH 7.0) [5] and sonicated for 10 s to obtain 10% (w/v) suspension. Cell membranes were then sedimented for 30 min at 100,000*g*.

All isolations were performed at 4 °C, mitochondria and cell membranes were stored at −80 °C. Protein concentration was measured according to [6].

### 2.3 Protein analysis by SDS PAGE and BNE/SDS PAGE

Mitochondrial pellets were suspended in MB2 buffer (1.5 M ε-aminocapronic acid, 150 mM Bis-tris, 0.5 mM EDTA, pH 7.0), solubilized with digitonin (8 g/g protein) for 15 min on ice and centrifuged for 20 min at 20,000*g*, 4 °C. Samples for BNE were prepared from supernatants by adding 1/20 volume of 5% SBG dye (Serva Blue G 250) in 750 mM ε-aminocapronic acid and 1/10 volume of 50% (v/v) glycerol. Solubilized tissue and cell mitochondria were analyzed by Bis-Tris BNE [7] on 5–12% polyacrylamide gradient gels. For two-dimensional separation by BNE/SDS PAGE, the stripes of BNE gel were incubated in 1% SDS and 1% 2-mercaptoethanol for 1 h and then subjected to SDS PAGE on a 10% slab gel [8].

Samples for SDS PAGE from 10% (w/v) cell suspension and solubilizates from cell membranes were prepared by adding the same volume of SLB2x lysis buffer and loaded on a 10% slab gel [8].

### 2.4 Western blot analysis

Proteins were transferred from the gels to PVDF membranes (Immobilon-P, Millipore) using semidry electroblotting. The membranes were blocked with 5% (w/v) non-fat dried milk in TBS (150 mM NaCl, 10 mM Tris, pH 7.5) for 1 h and incubated 2 h or overnight at 4 °C with primary antibodies diluted in TBS with 0.1% Tween-20. Monoclonal primary antibodies to the following enzymes of OXPHOS were used: SDHA (ab14715, Abcam), CORE1 (ab110252, Abcam), NDUFB6 (ab110244, Abcam), NDUFS3 (ab110246, Abcam), COX1 (ab14705, Abcam). The detection of the signals was performed with the secondary Alexa Fluor 680-labeled antibody (Life Technologies) using the Odyssey fluorescence scanner (LI-COR). Quantification of detected signals from SDS PAGE and BNE/SDS PAGE was carried out in Aida Image Analyzer program, version 3.21.

### 2.5 Doxycycline treatment of the cells

Experiment was performed as described in [9]. Briefly, fibroblasts (grown to 70% confluence in DMEM medium) were treated with 15 µg/ml doxycycline (DOX) for 7 days and then washed 3 times with PBS to withdraw DOX. Subsequently, the cells were collected at different time points (0, 6, 16, 24, 48, 72 and 96 h) after DOX removal. Weighed pellets of cells were stored at −80 °C. Two independent experiments of DOX inhibition in human and mouse fibroblasts were performed. The total COX1 signal for each given time point was quantified from 1D SDS PAGE using Aida Image Analyzer version 3.21 (Raytest) and normalized to SDHA signal.

## References

[1] Kovářová N., Pecina P., Nůsková H., Vrbacký M., Zeviani M., Mráček T., Viscomi C., Houštěk J. (1862). Tissue- and species-specific differences in cytochrome c oxidase assembly induced by SURF1 defects. Biochim. Biophys. Acta.

[2] Dell׳Agnello C., Leo S., Agostino A., Szabadkai G., Tiveron C., Zulian A., Prelle A., Roubertoux P., Rizzuto R., Zeviani M. (2007). Increased longevity and refractoriness to Ca(2+)-dependent neurodegeneration in Surf1 knockout mice. Hum. Mol. Genet..

[3] Pecina P., Čapková M., Chowdhury S.K., Drahota Z., Dubot A., Vojtíšková A., Hansíková H., Houšťková H., Zeman J., Godinot C., Houštěk J. (1639). Functional alteration of cytochrome c oxidase by SURF1 mutations in Leigh syndrome. Biochim. Biophys. Acta.

[4] Bentlage H.A., Wendel U., Schägger H., ter Laak H.J., Janssen A.J., Trijbels J.M. (1996). Lethal infantile mitochondrial disease with isolated complex I deficiency in fibroblasts but with combined complex I and IV deficiencies in muscle. Neurology.

[5] Wittig I., Braun H.P., Schägger H. (2006). Blue native PAGE. Nat. Protoc..

[6] Bradford M.M. (1976). A rapid and sensitive method for the quantitation of microgram quantities of protein utilizing the principle of protein–dye binding. Anal. Biochem..

[7] Schägger H., von Jagow G. (1991). Blue native electrophoresis for isolation of membrane protein complexes in enzymatically active form. Anal. Biochem..

[8] Schägger H., von Jagow G. (1987). Tricine-sodium dodecyl sulfate-polyacrylamide gel electrophoresis for the separation of proteins in the range from 1 to 100 kDa. Anal. Biochem..

[9] Moreno-Lastres D., Fontanesi F., García-Consuegra I., Martín M.A., Arenas J., Barrientos A., Ugalde C. (2012). Mitochondrial complex I plays an essential role in human respirasome assembly. Cell. Metab..

